# Psoriasis and Cardiovascular Disease: Lesion Beyond the Skin

**DOI:** 10.5935/abc.20190153

**Published:** 2019-08

**Authors:** Bruno Cesar Bacchiega

**Affiliations:** 1 Hospital de Câncer de Barretos, Barretos, SP - Brazil; 2 Santa Casa de Misericórdia de Barretos, Barretos, SP - Brazil

**Keywords:** Cardiovascular Diseases, Atherosclerosis, Inflammation, Risk Factors, Systemic Atherosclerotic Disease

Interesting to find an article^1^ that apparently belongs to the dermatology field in a cardiology journal. Why read about psoriasis? A disease that, for several years, many colleagues regarded as eminently skin-related and of benign course, especially in its plaque form. As it happens, epidemiological studies^2,3^ and an international registry^4^ revealed that the risk of cardiovascular (CV) events increased approximately 50% in the group of psoriatic individuals compared to the general population, markedly in younger patients.^5^ How to explain this situation? Systemic inflammation seems to be the "missing link" between CV diseases, neoplasms, and chronic systemic inflammatory diseases (e.g., psoriasis or rheumatoid arthritis).^6^

The interest of cardiology in this interdisciplinary theme is not new. In the past two decades, the understanding of atherosclerosis as a systemic vascular inflammatory disease and that sustained systemic inflammatory activity accelerates its physiopathological mechanisms has been consolidated. In 2003, Sattar et al*.*^7^ used rheumatoid arthritis as a physiopathological model to systematize the process of accelerated atherosclerosis. Systemic inflammation would increase the hepatic synthesis of C-reactive protein (CRP), induce lipolysis with the release of free fatty acids, and intensify insulin resistance and oxidation of low-density lipoproteins (LDL), culminating in endothelial dysfunction associated with increased expression of adhesion molecules and accelerating the atherosclerotic disease. In 2011, Boehnck et al.^8^ coined the term "psoriatic march" to describe possible mechanisms that could justify the increase in CV events in the physiopathological model of psoriasis. As expected, the foundation and steps of the process are extremely similar to those found in rheumatoid arthritis. The sustained systemic inflammation caused by psoriasis would increase the synthesis of CRP, vascular endothelial growth factor (VEGF), P-selectin, resistin, and leptin. Such proteins would be involved in raising insulin resistance, which ultimately would intensify the endothelial dysfunction and stimulate the expression of adhesion molecules. These steps reveal the importance of the subclinical atherosclerotic phase (endothelial dysfunction, vascular stiffness, and overexpression of vascular adhesion molecules) and biomarkers (CRP, leptin, and resistin) in this physiopathological process. In the article that prompted this short editorial, the authors compared arterial stiffness with pulse wave velocity (PWV), carotid intima-media thickness (IMT), metabolic syndrome data, and CRP levels in a cohort of psoriatic individuals and volunteers. The results reported were statistically significant and corroborated the hypothesis that psoriatic patients with intense disease activity are more prone to have metabolic syndrome, elevated CRP, and signs of subclinical atherosclerosis (increased IMT and arterial stiffness) compared to the control group.

The development of biomarkers to aid in the diagnosis, prognosis, and therapeutic follow-up of atherosclerotic disease is a reality. CRP and natriuretic peptides are well established for coronary artery disease and heart failure, respectively. Several biomarkers were studied specifically for psoriasis and risk of CV events: CRP, interleukin-6 (IL-6), leptin, and adiponectin. The results were similar to those of the general population, with increased CV risk in the presence of high CRP and IL-6 levels. Leptin presents conflicting findings, and adiponectin, as an adipokine with CV protective effects, is reduced in most studies.^9^ In addition, a new molecule might become a protagonist biomarker: GlycA - molecule analyzed by nuclear magnetic resonance spectrometry. This marker is correlated with the risk of coronary artery disease and vascular inflammation. In psoriatic patients, it was associated with both disease activity - estimated by the psoriasis area and severity index (PASI) - and risk of CV events, regardless of traditional risk factors, including CRP. Moreover, GlycA and vascular inflammation levels decreased in patients undergoing drug therapy with anti-tumor necrosis factor-alpha (anti-TNF-a),^10^ suggesting a correlation with therapeutic clinical response.

Given the importance of the theme, the American Academy of Dermatology^11^ and the European League Against Rheumatism (EULAR)^12^ have published recommendations on how to evaluate CV risk and make the necessary interventions to reduce such risks. Among the various scores that estimate CV risk factors, the EULAR group adopted the Systematic Coronary Risk Evaluation (SCORE), designed by the European Society of Cardiology. Unfortunately, no score or tool that estimates the probability of events can safely determine them in these high-risk populations (due to the chronic systemic inflammation). The dermatology guideline suggests increasing the estimated CV risk by 50% in psoriatic patients with active disease in >10% of the body surface or who have been indicated for phototherapy or systemic drug therapy to compensate. The EULAR group recommended the same procedure, proposing an adjustment of 1.5 times for patients with rheumatoid arthritis with extra-axial involvement, >10 years of disease, and presence of rheumatoid factor (RF) or serum anti-cyclic citrullinated peptide antibody (anti-CCP).

Another factor as important as risk stratification is the treatment. What are the possible drug interventions to control CV events in psoriasis? Reducing inflammation seems to be crucial to decrease CV diseases. In a cohort of psoriatic patients, the use of anti-TNF-a drugs decreased carotid IMT in men, and aortic stiffness in both genders.^13^ Even more impressive was the analysis of data collected by a Danish registry of psoriatic arthritis, in which the use of immunobiologicals and methotrexate reduced overall mortality.^14^ A meta-analysis helped to estimate the impact of anti-inflammatory therapies on the psoriatic population.^15^ The use of anti-TNF-a reduced major adverse cardiovascular events (MACE) - cardiovascular death, acute myocardial infarction (AMI), or non-fatal cerebrovascular accident (CVA) - robustly by 70%. Methotrexate led to a 19% drop in the risk of AMI and 28% in global events. On the other hand, treatment with selective COX-2 inhibitor non-steroidal anti-inflammatory drugs more than doubled the risk of CVA, while corticosteroid raised MACE by 62%.

Until now, the most debated randomized clinical study was the Canakinumab Anti-inflammatory Thrombosis Outcomes Study (CANTOS),^15^ which confirmed the hypothesis that the control of systemic inflammation reduces CV events in very high-risk patients (those who had AMI and even after optimized secondary therapy maintained high t-CRP levels). This study revealed that interleukin 1b blockade with a monoclonal antibody controlled the systemic inflammation and, additionally to the optimized treatment, decreased CV mortality, AMI, and CVA by 15% in a population with a high risk of recurrence of CV events. This fact opened doors for paradigmatic changes in the understanding of the physiopathology of the coronary artery disease and will contribute very significantly to the future development of new medicines, with targets unexplored until now.
